# Clinical uptake of an antigen-based approach to membranous nephropathy: a survey of general nephrologists and glomerular disease experts

**DOI:** 10.1007/s40620-025-02313-6

**Published:** 2025-06-11

**Authors:** Eibhlin Goggins, Andrew DeLaat, Bryce Barr, Jonathan Taliercio, Ali Mehdi, Georges Nakhoul, Brendan Bowman, Corey Cavanaugh

**Affiliations:** 1 Division of Nephrology, Center for Immunity, Inflammation, and Regenerative Medicine, University of Virginia, Charlottesville, VA USA; 2 Riverside Methodist Hospital, Columbus, OH USA; 3 Section of Nephrology, Department of Medicine, Max Rady College of Medicine, University of Manitoba, Winnipeg, Canada; 4 Department of Kidney Medicine, Cleveland Clinic Foundation, Cleveland, OH USA

**Keywords:** Membranous nephropathy (MN), M-type phospholipase A2 receptor (PLA2R), Kidney biopsy, Antigens

## Abstract

**Background:**

In recent years, there has been an emergence of new antigens discovered in membranous nephropathy (MN). Whether these antigens have impacted the approach to, and management of, MN patients undertaken by nephrologists is still unclear.

**Methods:**

We conducted a cross-sectional international survey pertaining to 13 antigens recently discovered in MN. The survey was distributed by the National Kidney Foundation, direct emails, and social media.

**Result:**

PLA2R, THSD7A, NELL1, and EXT1/2 testing were readily available while the most common response for other antigen testing was ‘Not Performed’ or ‘Unknown’. All respondents had tested for or treated PLA2R-positive MN. Of 79 respondents, only 12.7% had treated THSD7A, 15.2% for NELL1 and 6.3% for EXT1/2 positive MN. For PLA2R, THSD7A, and NELL1, a majority chose rituximab (75.4, 87.5, and 80.0%, respectively) as initial treatment, and would treat with immunosuppression before completing 6 months of conservative therapy. A majority of respondents would routinely or occasionally omit a kidney biopsy in the setting of positive serum anti-PLA2R antibodies, however, 27.5% would rarely do so. There was no clear consensus across respondents regarding the use of anti-PLA2R serum levels in determining remission.

**Conclusion:**

Although many new MN antigens have been discovered, there is limited availability of tests identifying these less common antigens. While the survey suggests potential for utilization of an antigen-tailored approach based on identified differences in screening and treatment practices, there remains a lag in the full adoption of this new information. Further progress in accessibility of antigen testing and research into antigen associations will enable a more individualized approach to the management of MN.

**Graphical abstract:**

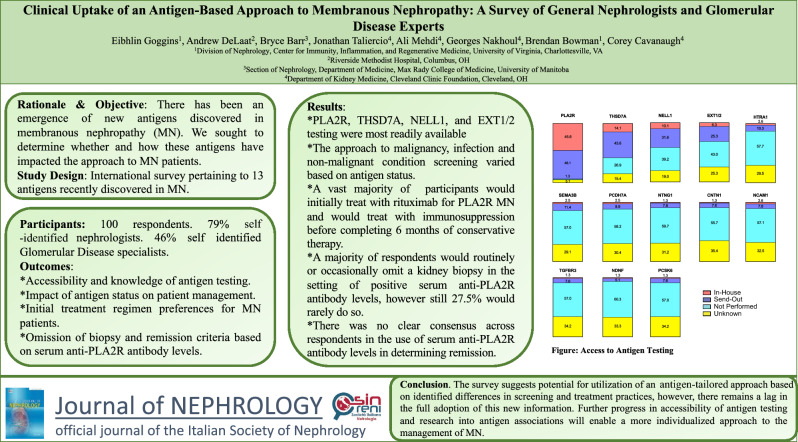

**Supplementary Information:**

The online version contains supplementary material available at 10.1007/s40620-025-02313-6.

## Introduction

Membranous nephropathy (MN), one of the most common causes of nephrotic syndrome in adults, is a pattern of glomerular injury characterized by immune complex deposition along the subepithelial region of the glomerular basement membranes. The original classification system divided MN into primary MN (idiopathic) and secondary MN (associated with systemic disease, drugs, and malignancy) which had important treatment implications. In 2009, the target antigen M-type phospholipase A2 receptor (PLA2R) and its circulating anti-PLA2R antibodies, were discovered [1]. This discovery revolutionized our understanding of the pathogenesis of MN as an antibody-mediated autoimmune disease. PLA2R-positive MN now accounts for approximately 50–80% of primary MN [2]. The classification system has been refined to PLA2R-positive or PLA2R-negative MN based on anti-PLA2R detection on the kidney biopsy specimen or in circulation. In 2014, a second target antigen, Thrombospondin type-I domain-containing 7A (THSD7A), was discovered and since then, numerous additional antigens have been identified alongside their respective autoantibodies [3]. Currently, target antigens can be identified in approximately 90% of individuals with MN. These discoveries have called into question the traditional classification of primary and secondary MN, especially given the detection of several target antigens in the presence of known ‘secondary’ causes. Instead, a two-step approach to diagnosis has been recommended, consisting of antigen identification, followed by ascertainment of a disease association, potentially guided by the target antigen [2].

These discoveries could enable a more personalized treatment approach with diagnostic and prognostic implications. While testing for some of these antigens can be performed at a few major centers, access to testing remains limited. Serum anti-PLA2R antibody monitoring has transformed our approach to treatment, as the antibody functions as a highly specific biomarker of disease activity [4–7]. Furthermore, with the development of the serum anti-PLA2R antibody test, MN can now be diagnosed without a biopsy [8–10]. This is reflected in the Kidney Disease: Improving Global Outcomes (KDIGO) 2021 clinical practice guideline for the management of glomerular diseases [10]. Circulating antibodies have been detected for all antigens included in this report, with the exception of EXT1/2 and TGFBR3 [2]. However, currently only the serum anti-PLA2R and anti-THSD7A antibody tests are commercially available. Thus, the diagnostic and prognostic value of testing serum levels of other antigens is unknown. However, if serum levels of additional antigens correlate with disease activity and prognosis, this could have a major impact on the future approach to MN.

We conducted a survey among practicing adult clinical nephrologists to assess if and how the new antigen information is currently utilized in clinical practice. More specifically, the aims were to assess (1) the awareness of nephrologists and the current availability of antigen testing (2) whether knowledge of antigen status has impacted the workup or treatment of patients and (3) how serum anti-PLA2R antibody testing is being used to guide diagnosis, remission status and biopsy decisions.

We anticipate that the development of commercially available assays and more research into antigen associations will lead to a change in clinical practice. Although many antigens have been discovered, testing for PLA2R, THSD7A, NELL1, and EXT1/2 is readily available at this time. Thus, we further analyzed questions related to these antigens to consider and use as an example of how antigen status could affect a physician’s approach to their patients. Finally, we further queried participants about their approach specifically to PLA2R-positive MN patients.

## Methods

### Survey development and analysis

An online survey was developed and piloted by the authors. Participants self-identified as nephrologists and were asked to provide their appointment, type of practice and years in practice. Participation was voluntary and any questions participants did not wish to answer could be left blank.

Survey questions referenced the following antigens:

CNTN1, contactin 1; EXT1/2, exostosin 1/2; NCAM1, neural cell-adhesion molecule 1; NDNF, neuron-derived neurotrophic factor; NELL1, neural epidermal growth factor–like protein 1; NTNG1, Netrin G1; PCDH7A, protocadherin 7A; PCSK6, proprotein convertase subtilisin/kexin type 6; PLA2R, M-type phospholipase A2 receptor; SEMA3B, semaphorin 3B; HTRA1, serine protease HTRA1; TGFBR3, transforming growth factor beta receptor 3; THSD7A, thrombospondin type-I domain-containing 7A.

General questions regarding access and awareness of testing as well as number of patients treated were included for each antigen. Additional antigen-specific questions could be answered only if the participant had managed a patient with the associated antigen. However, due to low numbers of patients treated, only questions pertaining to PLA2R, THSD7A, NELL1, and EXT1/2 were further analyzed. Descriptive analyses of percentages were reported where appropriate. Analysis was performed in Microsoft Excel and graphical representation of the data was completed in Prism®.

### Survey distribution

The survey was distributed by the National Kidney Foundation (NKF) through emails to their database of physicians starting in May 2024 and was open for 6 weeks. Physicians in the NKF database were sent an initial email and one follow up email. Additional emails were sent by the authors to nephrologists at various institutions throughout the country, requesting their participation and to distribute the survey to their nephrology department. Finally, a link to the survey was posted on the social media accounts of the authors.

## Results

### Survey participant characteristics

The survey was open for 6 weeks, from May through July 2024, with a total of 100 respondents, of whom 79 self-identified as practicing nephrologists. The 21 respondents that did not self-identify as practicing nephrologists were excluded from analyses.

Fifty-nine (74.7%) participants practiced in the United States with the remaining from 18 other countries. A majority of respondents identified as attending physicians either in an academic center (75.9%) or in private practice (16.5%), and 6.3% were nephrology fellows. Among them, 45.6% identified that they work in a glomerular disease clinic and/or specialize in glomerular disease. Additional questions related to PLA2R, THSD7A, NELL1, and EXT1/2 were further analyzed. Because only few respondents had managed patients positive for the remaining antigens, these were not analyzed further. The demographic and practice characteristics of the respondents are summarized in Supplemental Table 1.

### Access to antigen testing

**Fig. 1:**
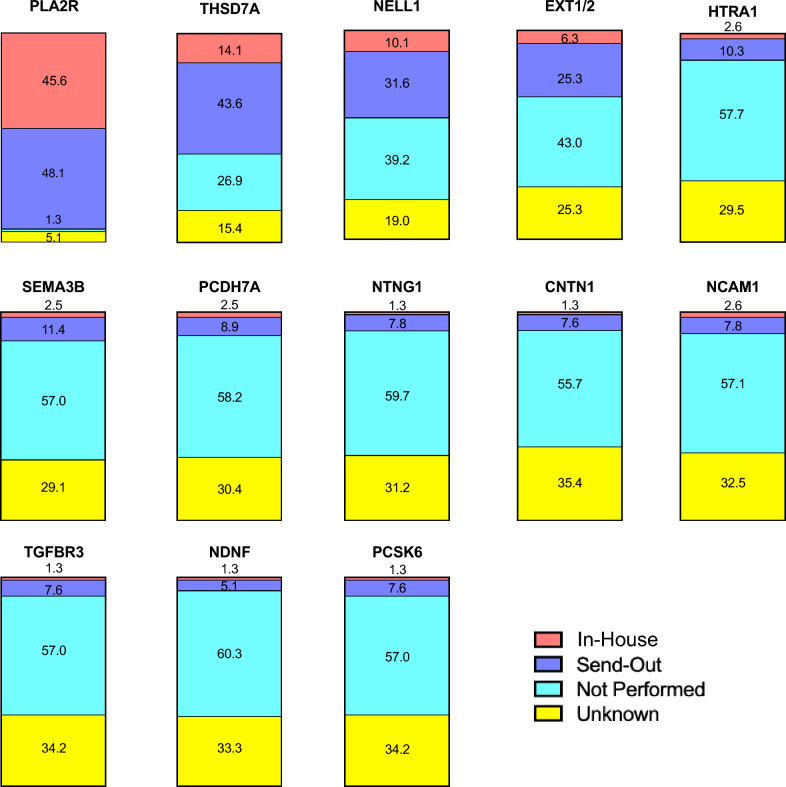
Access to antigen testing. Participants were asked whether the primary pathology lab they send biopsies to performs testing for the antigen in-house or send-out, whether it is not performed, or whether the availability of testing is unknown. A table of values is provided in Supplemental Table 2

In all countries, respondents reported access to PLA2R testing either performed in-house (45.6%) or by a send-out mechanism (48.1%). For THSD7A, 14.1% of respondents reported having testing available in-house and 43.6% by send-out. This was followed by NELL1, for which 10.1% of respondents reported having testing available in-house and 31.6% by send-out. Finally, for EXT1/2, 6.3% of respondents reported having testing available in-house and 25.3% by send-out. For all other antigens, access to in-house or send-out testing ranged from only 6.4 to 13.9%, and many indicated that access to availability of testing was unknown (Fig. 1, Supplemental Table 2).

**Fig. 2:**
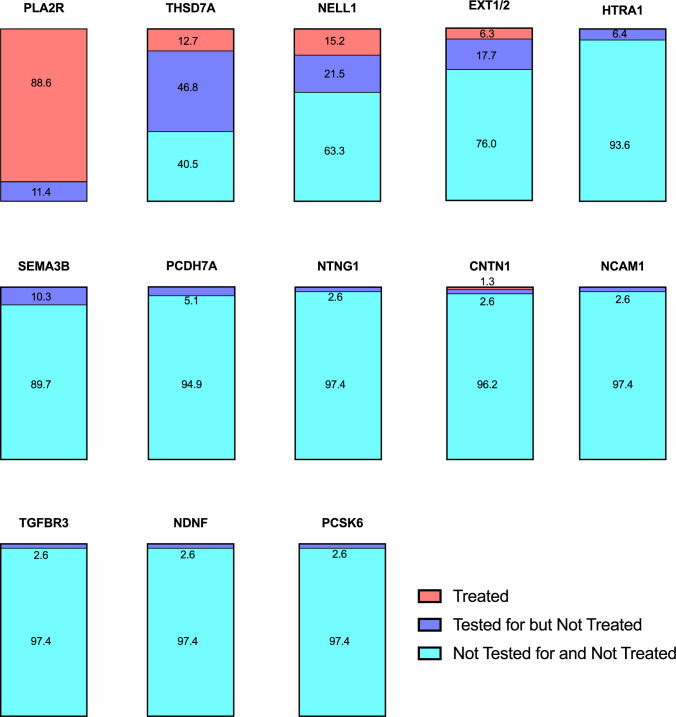
Testing and treatment of MN patients by antigen status. Participants were asked whether they had tested for and/or treated MN patients positive for each antigen

Nephrologists were then asked whether they had tested for or treated patients positive for each of the antigens. All respondents had tested for or treated patients positive for PLA2R. Of the other antigens, nephrologists had treated THSD7A (12.7%), NELL1 (15.2%), EXT1/2 (6.3%) positive patients and 1 respondent had treated a contactin-positive patient. Among nephrologists, 46.8%, 21.5% and 17.7% had not treated but had tested for THSD7A, NELL1, and EXT1/2, respectively. Less than 11% of nephrologists had tested for each of the remaining antigens (Fig. 2).

### Evaluation for secondary causes and associations

**Fig. 3:**
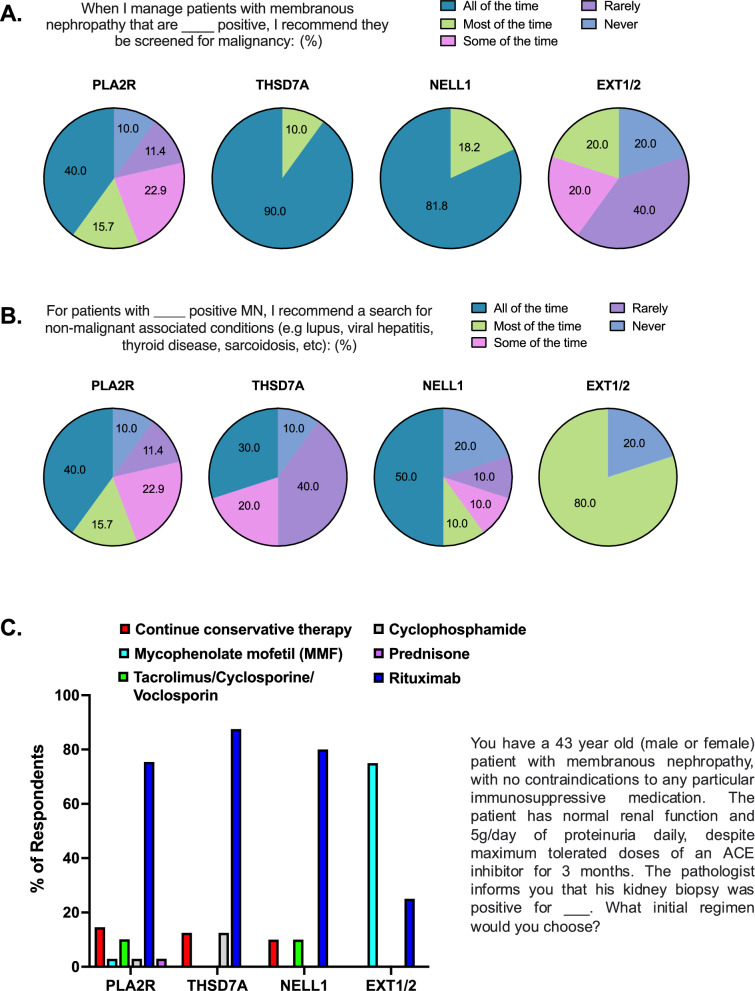
Screening and treatment practices for patients presenting with MN according to antigen status. **A** Participants were asked to state how often they screen for malignancy in MN patients by antigen status. **B** Participants were asked to state how often they screen for non-malignant associated conditions in MN patients by antigen status. **C** For each antigen, participants were asked what treatment option they would initiate for the MN patient in the prompt. Participants could select multiple answers (*n* = 69, 8, 10, and 4 for PLA2R, THSD7A, NELL1, and EXT1/2, respectively)

Membranous nephropathy is associated with various diseases including malignancy, autoimmune diseases, and infections [2]. Included in Fig. 3A–B are the percentages of participants who would screen for these associations for PLA2R, THSD7A, NELL1, and EXT1/2. Supplemental Tables 3–5 are the specific laboratory tests that would be performed for PLA2R-positive MN patients. Similar trends in specific tests performed were obtained for THSD7A, NELL1, and EXT1/2, however, the small number of respondents and questions with multiple answer choices limited these data.

Regarding malignancy workup, a vast majority of participants would perform screening in THSD7A (90%) and NELL1 (81.8%) positive patients (Fig. 3A). For PLA2R-positive patients, the results were dispersed, however 78.6% would screen for malignancy some, most, or all of the time. Notably, for EXT1/2, 20% and 40% said they would never or rarely screen for malignancy, respectively. For the specific tests performed, colonoscopy, mammogram, Prostate Specific Antigen (PSA), PAP smear/HPV testing, and Computed Tomography (CT) scan of the chest were the tests most often performed for PLA2R-positive MN (Supplemental Table 3).

Participants were then surveyed about their workup for non-malignant conditions (Fig. 3B). Notably, 80% would screen for other conditions, including lupus, most of the time in EXT1/2-positive patients. On the other hand, the results for PLA2R, THSD7A, and NELL1 were dispersed across all answer choices. Regarding laboratory tests related to autoimmune and other conditions, for PLA2R-positive MN, ANA, Complement (C3/C4), Serum Protein Electrophoresis, Anti-Double Stranded DNA, and Hemoglobin A1C were most commonly obtained, with other tests having scattered results across respondents (Supplemental Table 4).

Finally, MN has been associated with a variety of infections, particularly HIV, Hepatitis B and C, as well as a few case reports of others such as schistosomiasis [11] or filariasis [12]. We assessed whether physicians screen broadly for multiple infections or focus on ones most associated with MN. As shown in Supplemental Table 5, the most commonly screened for infections in PLA2R-positive MN, were Hepatitis B and C, HIV, followed by syphilis, with others tested for by less than 5% of respondents. Of note, 20.3% said they do not routinely test for infections.

### Membranous nephropathy treatment practices

In this survey, each nephrologist was asked to indicate their preferred initial treatment regimen for a patient with MN positive for PLA2R, THSD7A, NELL1, or EXT1/2. Participants could also choose to continue conservative management. Specifically, participants were asked the following, regarding an MN patient with no contraindications to any immunosuppressive medications:

*“The patient has normal renal function and 5g/day of proteinuria, despite maximum tolerated doses of an ACE inhibitor for 3 months. The pathologist informs you that his kidney biopsy was positive for __. What initial regimen would you choose?”*

For PLA2R, THSD7A, and NELL1, a large majority chose rituximab (75.4, 87.5, and 80.0%, respectively) (Fig. 3C), while 14.5, 12.5, and 10%, respectively, chose to continue conservative management in this moderate risk clinical scenario.

On the other hand, for EXT1/2, mycophenolate mofetil was chosen by most respondents (75%), although responses were limited. Those that selected mycophenolate mofetil also stated that the antigen affected their treatment decision.

For nephrologists who had treated MN patients with co-existing malignancy, all respondents would ‘Treat the malignancy first and monitor the renal response’ in the setting of THSD7A- and NELL1-positive MN. For PLA2R-positive MN, 67.4% would treat the malignancy first, while 32.6% would treat simultaneously with immunosuppressive therapy specific for PLA2R MN. None of the participants would treat the MN first regardless of antigen status.

### Management of M-type phospholipase A2 receptor (PLA2R) membranous nephropathy

In this survey, in addition to the questions for all antigens, participants were asked additional PLA2R-specific questions.

**Fig. 4:**
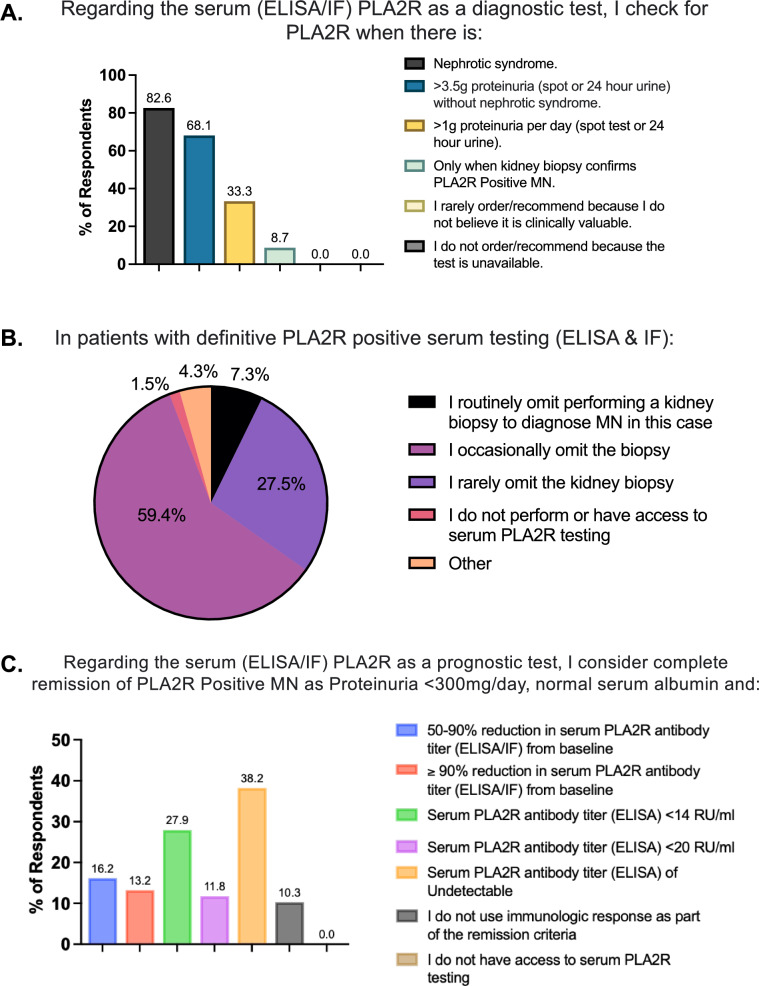
Serum anti-PLA2R antibody testing utilization. **A** *Diagnostic value of the serum anti-PLA2R antibody test*: Participants were surveyed for their use of the ELISA/IF serum anti-PLA2R antibody test as a diagnostic test. Participants could select multiple answers. Values above each bar correspond to the respective percentage. **B** *Omission of the kidney biopsy:* Participants were asked whether and how often they omit obtaining a kidney biopsy in patients with a positive serum anti-PLA2R antibody test. **C** *Use of serum anti-PLA2R antibody test in remission and prognosis:* Participants were surveyed for their use of ELISA/IF serum anti-PLA2R antibody levels in their remission criteria. Participants could select multiple answers. Values above each bar correspond to percentages

Participants were first asked to state when they would check serum anti-PLA2R antibody levels as a diagnostic test (Fig. 4A). A majority of respondents (82.6%) would perform serum anti-PLA2R antibody testing when nephrotic syndrome is present or when the patient has > 3.5 g proteinuria (spot or 24-h urine) without nephrotic syndrome (68.1%). Overall 33.3% would order the serum anti-PLA2R antibody test when there is > 1 g proteinuria per day (spot test or 24 h urine), and 8.7% would check only after a kidney biopsy has confirmed PLA2R-positive MN.

Participants were then surveyed to determine whether a positive serum anti-PLA2R antibody test would affect their decision to obtain a kidney biopsy (Fig. 4B). Regarding a patient with definitive anti-PLA2R-positive serum testing (ELISA and IF), a majority of respondents (66.7%) reported that they routinely or occasionally omit the kidney biopsy (7.3% and 59.4%, respectively). However, 27.5% would rarely omit the biopsy regardless of serum testing.

Finally, participants were asked if and whether they use serum anti-PLA2R antibody levels to determine remission of PLA2R-positive MN (Fig. 4C). Answer choices could be based on a percent decline from the patient’s baseline and/or by the absolute value. Notably, there was a large spread across all answer choices with a small percentage (10.3%) stating that they do not use immunologic response as part of the remission criteria.

## Discussion

There has been an emergence of new antigens, along with their disease associations, discovered in membranous nephropathy in recent years. The objective of this survey was to assess whether the discovery of these antigens has impacted the approach to, and treatment of, MN patients. This survey, distributed to nephrologists internationally, included questions pertaining to 13 recently discovered antigens and additional questions regarding the use of serum anti-PLA2R antibody levels. Notably, the results revealed that few nephrologists have experience in the testing for, or treatment of, non-PLA2R-positive MN, even amongst glomerular disease experts. The recent Mayo Clinic consensus report on MN proposed an antigen-based classification scheme [2], however, this survey suggests that most nephrologists do not yet have access to antigen and antibody testing in order to adopt this approach.

MN is associated with a variety of diseases including malignancy, autoimmune diseases, and infections [2]. Thus, the evaluation could be broad without more detailed patient information and may be dependent on local practice patterns and socioeconomic status. The survey identified differences in the workup and screening of patients based on antigen status for PLA2R, THSD7A, NELL1, and EXT1/2. For example, nephrologists appear to adopt a more aggressive malignancy workup for the antigens associated with malignancy, NELL1 and THSD7A, but not for EXT1/2 which has been associated with autoimmune diseases. This suggests that nephrologists are considering the described antigen associations in their diagnostic workup. Additionally, this may suggest that, once routine testing for more antigens becomes available, we may be able to tailor the workup of patients and perform a more targeted evaluation. This could prevent unnecessary testing and a miss or delay in diagnosis. For example, in addition to malignancy, NELL1 has numerous secondary associations [13], and was recently found to be associated with thiol-containing medications including lipoic acid, bucillamine, and tiopronin, as well as mercury-containing traditional indigenous medicines [14–17]. Importantly, a majority of patients achieve remission upon discontinuation of the offending agent without the need for immunosuppression [14, 17]. Therefore, in these cases, identification of the target antigen and an awareness of its association with these agents is crucial in providing proper care. Without this, the agents may not be identified during the patient workup, leading to ongoing exposure to the offending agent and inappropriate treatment of patients who otherwise have excellent prognoses. Similarly, contactin 1 (CNTN1), discovered in 2020, appears to be specifically associated with chronic inflammatory demyelinating polyradiculoneuropathy [18]. Ideally, if a patient was determined to be CNTN1-positive, they would then be evaluated for chronic inflammatory demyelinating polyradiculoneuropathy. Otherwise, as a less common diagnosis, chronic inflammatory demyelinating polyradiculoneuropathy may not be included in the workup of a patient presenting with MN, leading to a missed or delayed diagnosis.

While improved classification of patients is important, critical is whether this ultimately affects our approach, leading to improved and personalized management of MN patients. Before the 21st century, there was only minimal improvement in outcomes for MN patients. Treatment relied on steroids, immunosuppressive agents and cyclosporine, all of which carried significant side effects. The Ponticelli regimen, first described in 1984, and the later modified Ponticelli regimen, were effective but highly toxic [19–21]. Other agents such as calcineurin inhibitors were less toxic but associated with significant relapse risk.

The survey revealed rituximab to be the dominant choice in immunosuppressive therapy amongst participants. In 2002, the first report of MN patients treated with rituximab was published [22]. Since this report, the treatment of MN has evolved considerably, with additional pharmaceutical options becoming available. In 2019, the MENTOR trial firmly established rituximab as superior to calcineurin inhibitors [23]. However, the decision of when to choose rituximab or a cyclophosphamide-based regimen for severe to very severe MN remains controversial, as recent trial data comparing the regimens were inconclusive [24]. In a multinational survey of the South Asian-Pacific region of mostly senior nephrologists, the combination of corticosteroid and cyclophosphamide was the preferred first line therapy, while rituximab monotherapy was preferred in < 10% of respondents as first line [25]. Similarly, in a recent survey conducted amongst nephrologists in Japan, a majority indicated that corticosteroid monotherapy was the most common first line choice for MN (240 of 403, 59.6%) [26]. Similar results were seen in the 2019 analysis of the CUREGN cohort, in which 1 in 5 treated patients received glucocorticoid monotherapy [27].

It is important to note that rituximab and other immunosuppressive agents have many side effects and many patients with MN develop spontaneous remission without the need for any immunosuppression [10]. Thus KDIGO emphasizes that, in patients without risk factors or complications, conservative therapy (ACEi/ARB) should first be employed for 6 months before initiating immunosuppression [10]. Therefore, we asked participants about their treatment regimen for a moderate risk patient after 3 months on an ACEi (Fig. 3C). Interestingly, despite it being a patient with no additional risk factors and the timeframe being before the recommended 6-month ‘wait and see’ period, only a small minority chose to continue conservative management. For PLA2R, THSD7A, and NELL1, a large majority chose rituximab (75.4, 87.5, and 80.0%, respectively). Similarly, in the aforementioned survey conducted amongst nephrologists in Japan, it was found that 33.5% would not wait before initiating immunosuppression in MN patients with > 6 g/day of proteinuria even without other risk factors [26]. In the case of PLA2R MN, one explanation for the lack of adherence to the timeframe guidelines may be that nephrologists can monitor serum anti-PLA2R antibody levels, allowing for earlier therapeutic decisions, as these have been shown to be predictive of longer-term outcomes [28].

For EXT1/2, mycophenolate mofetil was chosen by most respondents (75%), although responses were limited. Notably, those that selected mycophenolate mofetil also stated that the antigen affected their treatment decision. EXT1/2 were identified as novel antigens in MN associated with autoimmune disease, particularly lupus nephritis (LN), in which it was found in 32.6% of LN Class V patients [29, 30]. That respondents chose mycophenolate mofetil specifically for EXT1/2 patients suggests that this association has made an impact in treatment decisions. EXT1/2-positive LN patients have distinct clinical features, notably favorable outcomes despite high proteinuria. Therefore, it is plausible that an even more specific treatment approach to this subgroup of LN patients could be developed.

The 2009 discovery of circulating autoantibodies against PLA2R and development of the serum anti-PLA2R antibody test revolutionized our understanding of, and approach to, MN. Although kidney biopsy has been considered the gold standard for diagnosing MN, with the use of serum anti-PLA2R antibody levels, MN can be diagnosed without a biopsy. Bobart et al. found that among patients with MN and positive serum anti-PLA2R antibodies, a negative workup for secondary causes of MN, and normal kidney function, the addition of a kidney biopsy only confirmed the MN diagnosis without influencing clinical management [9]. Similar studies have suggested that kidney biopsy may be unnecessary in PLA2R-positive MN [8]. Accordingly, the 2021 KDIGO guideline states that a “kidney biopsy is not required to confirm the diagnosis of membranous nephropathy (MN) in patients with nephrotic syndrome and a positive anti-PLA2R antibody test” [10]. Thus, participants were queried regarding their use of the kidney biopsy and antibody testing. While a majority of respondents stated they routinely or occasionally omit the kidney biopsy, still 27.5% answered that they would rarely omit the biopsy regardless of serum testing.

Finally, serum anti-PLA2R antibody levels are known to correlate with disease activity, thus providing both diagnostic and prognostic value [4–7]. While this is useful in monitoring anti-PLA2R levels, it is unclear whether nephrologists follow any guidelines to determine immunologic remission. KDIGO states that a serum value of less than 2 RU/ml by ELISA or negative immunofluorescence test should be used to determine complete immunologic remission [10]. The survey identified a large spread of criteria used amongst participants which may suggest that each nephrologist uses their own judgment in considering serum anti-PLA2R antibody levels in remission criteria (Fig. 4C).

This survey relied on self-reported data and was limited by its small sample size, preventing our ability to draw statistically significant conclusions. Additionally, while the survey was distributed internationally and 19 countries were represented, a majority of respondents were located in the United States and thus the survey may not reflect global treatment practices. The cost effectiveness of testing is not yet known and may depend upon geographic differences in accessibility. Finally, at this point in time, there is a paucity of information in the literature and thus we could not assess more detailed uses of many of the antigens other than PLA2R, THSD7A, NELL1, and EXT1/2, nor could we assess long-term patient outcomes. However, this further underscores the need for additional research and progress in developing accessible tools in antigen testing especially as new antigens are discovered. Future studies can then be undertaken to clarify their value with respect to diagnostic and therapeutic decisions.

## Conclusion

There has been considerable progress made over the past few years in identifying novel antigens in MN. However, this survey identified that nephrologists have limited access to testing and exposure to antigens other than PLA2R, THSD7A, NELL1, and EXT1/2 even amongst glomerular disease experts. This survey assessed (1) the awareness of nephrologists and current availability of antigen testing (2) whether knowledge of antigen status has impacted the workup or treatment of patients and (3) how serum anti-PLA2R antibody testing is being used to guide diagnosis, remission status and kidney biopsy decisions.

Serum anti-PLA2R antibody testing is accessible, familiar and used routinely for diagnostic and prognostic purposes. However, despite data supporting the efficacy in omission of kidney biopsy, 27.5% of those surveyed would ‘rarely’ omit a kidney biopsy, regardless of serum testing. Additionally, despite KDIGO guideline recommendations that, before considering immunosuppression, conservative therapy should be tried for 6 months in moderate risk patients, a majority of respondents chose rituximab as first line therapy in PLA2R-positive MN at month 3.

Finally, this survey identified differences in screening and treatment practices for PLA2R, THSD7A, NELL1, and EXT1/2 positive patients which suggests that currently known antigen associations are being taken into account. In future, we predict that, with further progress in the accessibility of novel antigen testing, it will be possible to design more tailored approaches to the workup and treatment of individual MN patients.

## Supplementary Information

Below is the link to the electronic supplementary material.

Supplementary file1 (DOCX 40 KB)

## Data Availability

Data are available upon request.
