# Supplementary material for: Clinical uptake of an antigen-based approach to membranous nephropathy: a survey of general nephrologists and glomerular disease experts
Source: J Nephrol. 2025 Jun 11;38(7):1889–900. doi: 10.1007/s40620-025-02313-6 (PMC12484325; doi:10.1007/s40620-025-02313-6)
Supplement: Supplementary file 1 — Supplementary file1 (DOCX 40 KB) [file 40620_2025_2313_MOESM1_ESM.docx]

**Supplemental Table 1. Self-Reported Demographic and Practice-Related Characteristics of Participants (%)**

|  | Total  (n = 79) | GD Clinic/  Specialist  (n = 36) | Non-GD  (n = 43) |
| --- | --- | --- | --- |
| Appointment |  |  |  |
| Fellow  Attending (Private Practice)  Attending (Academic)  Other | 6.3  16.5  75.9  1.3 | 11.1  11.1  77.8  0 | 2.4  21.4  76.2  2.3 |
| Years in Practice |  |  |  |
| 1-5  6-10  11-20  > 20 | 29.1  20.3  27.8  22.8 | 30.6  19.4  27.8  22.2 | 27.9  20.9  27.9  23.2 |
| Country |  |  |  |
| United States  Other | 74.7  25.3 | 63.9  36.1 | 81.8  18.2 |
| Area |  |  |  |
| Rural Area  Urban Area  Suburban Area | 9.0  69.2  21.8 | 2.8  83.3  13.9 | 14.3  57.1  28.6 |
| Specialty* |  |  |  |
| General Adult Nephrology Transplant Nephrology  Interventional Nephrology  Onconephrology  Dialysis  Pathology  Other | 89.9  12.7  5.1  6.3  27.9  2.5  8.9 | 88.9  8.6  0  3.0  15.6  3.2  116.7 | 88.6  15.9  9.3  9.5  41.5  2.5  2.3 |

Glomerular Disease (GD)

*More than one answer accepted

**Supplemental Table 2. Access to Antigen Testing.** Participants were asked whether the primary pathology lab they send biopsies to performs testing for the antigen In House or Send-out, whether it is not performed, or whether the availability of testing is unknown (%). Percentages are reported based on the number of respondents for each antigen. Missing data, where present, is the percentage of respondents that did not provide an answer for each antigen out of the total n in each column The data is represented graphically in Figure 1.

|  | Total  (n = 79) | GD Clinic/  Specialist  (n = 36) | Non-GD  (n = 43) |
| --- | --- | --- | --- |
| PLA2R  In House  Send-Out  Not Performed  Unknown  Missing | 45.6  48.1  1.3  5.1  0 | 52.8  41.7  2.8  2.8  0 | 39.5  53.5  0  7.0  0 |
| THSD7A  In House  Send-Out  Not Performed  Unknown  Missing | 14.1  43.6  26.9  15.4  1.3 | 16.7  47.2  27.8  8.3  0 | 11.9  40.5  26.2  21.4  2.3 |
| NELL1  In House  Send-Out  Not Performed  Unknown  Missing | 10.1  31.6  39.2  19.0  0 | 11.1  44.4  36.1  8.3  0 | 9.3  20.9  41.9  27,9  0 |
| EXT1/2  In House  Send-Out  Not Performed  Unknown  Missing | 6.3  25.3  43  25.3  0 | 8.3  36.1  47.2  8.3  0 | 4.7  16.3  39.5  39.5  0 |
| HTRA1  In House  Send-Out  Not Performed  Unknown  Missing | 2.6  10.3  57.7  29.5  1.3 | 0  11.1  75.0  13.9  0 | 4.8  9.5  42.9  42.9  2.3 |
| SEMA3B  In House  Send-Out  Not Performed  Unknown  Missing | 2.5  11.4  57.0  29.1  0 | 0  16.7  72.2  11.1  0 | 4.7  7.0  44.2  44.2  0 |
| PCDH7A  In House  Send-Out  Not Performed  Unknown  Missing | 2.5  8.9  58.2  30.4  0 | 0  8.3  77.8  13.9  0 | 4.7  9.3  41.9  44.2  0 |
| NTNG1  In House  Send-Out  Not Performed  Unknown  Missing | 1.3  7.8  59.7  31.2  2.6 | 0  8.3  77.8  13.9  0 | 2.4  7.3  43.9  46.3  4.6 |
| CNTN1  In House  Send-Out  Not Performed  Unknown  Missing | 1.3  7.6  55.7  35.4  0 | 0  5.6  72.2  22.2  0 | 2.3  9.3  41.9  46.5  0 |
| NCAM1  In House  Send-Out  Not Performed  Unknown  Missing | 2.6  7.8  57.1  32.5  2.6 | 0  5.6  75.0  19.4  0 | 4.9  9.8  41.5  43.9  4.6 |
| TGFBR3  In House  Send-Out  Not Performed  Unknown  Missing | 2.3  7.6  57.0  34.2  0 | 0  8.3  72.2  19.4  0 | 2.3  7.0  44.2  46.5  0 |
| NDNF  In House  Send-Out  Not Performed  Unknown  Missing | 1.3  5.1  60.3  33.3  1.3 | 0  2.8  75.0  19.4  2.8 | 2.3  7.0  46.5  44.2  0 |
| PCSK6  In House  Send-Out  Not Performed  Unknown  Missing | 1.3  7.6  57.0  34.2  0 | 0  5.6  72.2  22.2  0 | 2.3  9.3  44.2  44.2  0 |

Glomerular Disease (GD)

**Supplemental Table 3. Malignancy Screening Tests in the Workup of PLA2R Positive MN Patients**

|  | (%) |
| --- | --- |
| Colonoscopy | 68.1 |
| Mammogram (Female) | 53.6 |
| Prostate Specific Antigen (Male) | 50.7 |
| Pap Smear/HPV Testing (Female) | 42.0 |
| CT Scan of Chest | 30.4 |
| CT Abdomen/Pelvis | 26.1 |
| X-Ray of Chest | 23.2 |
| Kidney Ultrasound | 21.7 |
| Refer to PCP for Cancer Screening | 20.3 |
| Fecal Occult Blood Testing | 11.6 |
| I would Not Recommend Malignancy Screening | 7.2 |
| 18F-FDG PET/CT | 2.9 |
| Cystoscopy and Cytology | 1.4 |
| Testicular Ultrasound (Male) | 1.4 |
| Thyroid Ultrasound | 0.0 |

**Supplemental Table 4. Non-Malignant Associated Condition Screening Tests in the Workup of PLA2R Positive MN Patients**

|  | (%) |
| --- | --- |
| ​​ANA | 74.1 |
| ​​Complement (C3/C4) | 67.2 |
| ​​​Serum Protein Electrophoresis | 62.1 |
| ​​Anti-Double Stranded DNA | 48.3 |
| ​​Hemoglobin A1C | 39.7 |
| ​​​Extractable Nuclear Antigen (ENA i.e. Scleroderma, Sjogrens Related Antibodies) | 22.4 |
| ​​​Rheumatoid Factor | 15.5 |
| ​Anti-Ro/La SSA/SSB | 13.8 |
| ​​Anti-MPO | 12.1 |
| ​​Anti-PR3 | 10.3 |
| ​​Anti-GBM | 10.3 |
| ​IgG4 | 8.6 |
| ​​Anti-Thyroid Antibodies (Anti-TPO) | 5.2 |
| ​​ACE Level | 5.2 |

**Supplemental Table 5. Infection Screening Tests in the Workup of PLA2R Positive MN Patients**

|  | (%) |
| --- | --- |
| Hepatitis B | 78.3 |
| Hepatitis C | 71.0 |
| HIV | 66.7 |
| Syphilis | 37.7 |
| CMV | 4.3 |
| EBV | 2.9 |
| Plasmodium Malaria | 1.4 |
| Filariasis | 1.4 |
| Schistosomiasis | 1.4 |
| Mycobacterium leprosy | 0.0 |
| I do not routinely test for infections | 20.3 |
